# Role of GABA_A_ inhibition in modulation of pyramidal tract neuron activity during postural corrections

**DOI:** 10.1111/j.1460-9568.2007.05413.x

**Published:** 2007-03

**Authors:** Zinaida A Tamarova, Mikhail G Sirota, Grigori N Orlovsky, Tatiana G Deliagina, Irina N Beloozerova

**Affiliations:** 1 Barrow Neurological Institute, St Joseph's Hospital and Medical Center350 West Thomas Road, Phoenix, AZ 85013, USA; 2 Bogomoletz Institute of PhysiologyKiev, Ukraine; 3 Karolinska InstituteSE-17177, Stockholm, Sweden

**Keywords:** balance, bicuculline, cat, gabazine, iontophoresis, motor cortex

## Abstract

In a previous study we demonstrated that the activity of pyramidal tract neurons (PTNs) of the motor cortex is modulated in relation to postural corrections evoked by periodical tilts of the animal. The modulation included an increase in activity in one phase of the tilt cycle and a decrease in the other phase. It is known that the motor cortex contains a large population of inhibitory GABAergic neurons. How do these neurons participate in periodic modulation of PTNs? The goal of this study was to investigate the role of GABA_A_ inhibitory neurons of the motor cortex in the modulation of postural-related PTN activity. Using extracellular electrodes with attached micropipettes, we recorded the activity of PTNs in cats maintaining balance on a tilting platform both before and after iontophoretic application of the GABA_A_ receptor antagonists gabazine or bicuculline. The tilt-related activity of 93% of PTNs was affected by GABA_A_ receptor antagonists. In 88% of cells, peak activity increased by 75 ± 50% (mean ± SD). In contrast, the trough activity changed by a much smaller value and almost as many neurons showed a decrease as showed an increase. In 73% of the neurons, the phase position of the peak activity did not change or changed by no more than 0.1 of a cycle. We conclude that the GABAergic system of the motor cortex reduces the posture-related responses of PTNs but has little role in determining their response timing.

## Introduction

Pyramidal tract neurons (PTNs) have been the focus of many studies of motor cortex activity during motor tasks (e.g. Evarts, 1968; Fetz & Finocchio, 1972; Conrad *et al*., 1977; Martin & Ghez, 1985; Georgopoulos *et al*., 1988, 1992; Mountcastle, 1980; Scott & Kalaska, 1995, 1997; Kakei *et al*., 2003). These studies provide fundamental knowledge about the involvement of these cells and the motor cortex generally in the control of different motor behaviours. Considerable effort has been aimed at characterising the intracortical networks that underlie this involvement (e.g. Birt *et al*., 2003; Jacobs & Donoghue, 1991; Matsumura *et al*., 1991, 1992; Castro-Alamancos *et al*., 1995; Kubota, 1996; Connors & Telfeian, 2000; Schneider *et al*., 2002; Capaday, 2004). It has been shown that gamma-aminobutyric acid (GABA) plays an important role in regulating motor-related activity in the motor cortex. When the motor cortex was deprived of GABA by injection of bicuculline, a GABA_A_ receptor antagonist, manual dexterity in monkeys was severely impaired (Matsumura *et al*., 1991). When bicuculline was applied iontophoretically onto individual neurons in the monkey motor cortex, their activity during lever pressing increased (Matsumura *et al*., 1992). Electrical stimulation studies in the rat motor cortex showed that bicuculline broadened output motor fields (Jacobs & Donoghue, 1991). In chloralose-anaesthetized cats, Capaday & Rasmusson (2003) found that bicuculline expands somatosensory receptive fields of cells in the motor cortex. Also in anaesthetized cat preparations, data were obtained suggesting that selective GABA_A_ inhibition and disinhibition may functionally link different neuronal groups of the motor cortex in useful motor synergies (Schneider *et al*., 2002). To further understand of the role of GABA_A_-mediated influences in cortical motor-related activity, however, different motor behaviours in awake animals need to be studied.

**Fig. 1:**
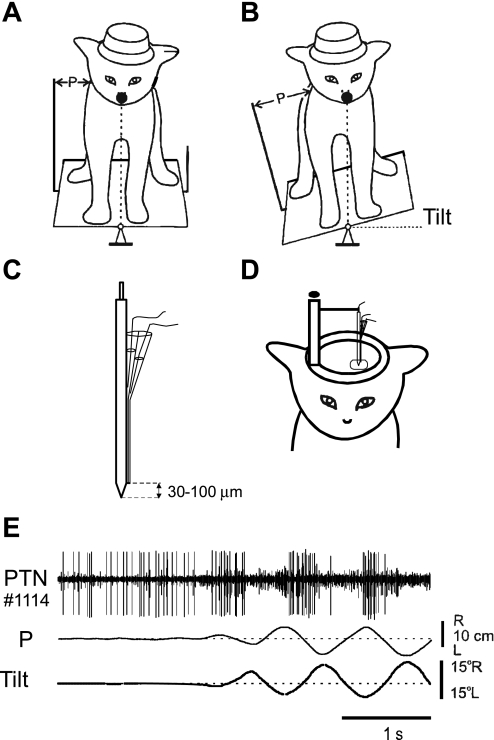
Experimental design. (A) The cat stood on a platform. A mechanical sensor P measured displacements of the body in relation to the platform (postural corrections). (B) When the platform was tilted to the right, the cat kept the dorsal-side-up orientation by extending the limbs on the right side. A mechanical sensor measured the angular displacement of the platform (Tilt). (C) A system for recording the activity of PTNs and for iontophoretic application of gabazine or bicuculline. A two- or three-channel glass pipette was attached to an extracellular microelectrode. Not shown to scale. (D) The electrode and pipette were inserted into the motor cortex by a miniature microdrive attached to the head. (E) PTN activity was recorded along with platform tilts and postural corrections; R, right; L, left.

In quadrupeds, the dorsal-side-up body orientation is maintained by a closed-loop control system that performs a highly automatic, nonvoluntary motor function (Magnus, 1924; Bard & Macht, 1958). In this postural system the generation of a corrective motor response is based on various sensory feedback signals (reviewed in Horak & Macpherson, 1996; Macpherson *et al*., 1997; Massion, 1998; Deliagina & Orlovsky, 2002). Although the basic nervous mechanisms for feedback postural control reside in the brainstem, cerebellum and spinal cord, participation of the motor cortex in the control system has been demonstrated in lesion studies, by transcranial magnetic stimulation, and by intracortical electrical stimulation (e.g. Gahery & Nieoullon, 1978; Ioffe, 1973; Massion, 1979; Ioffe *et al*., 1988, 2002; Lavoie *et al*., 1995; Latash *et al*., 2003; Solopova *et al*., 2003). Using a single-cell recording technique in cats and rabbits, we have shown that the activity of descending efferent neurons of layer V of the motor cortex, including pyramidal tract neurons, is modulated during postural corrections (Beloozerova *et al*., 2003, 2005). When the supporting platform was tilted periodically, the activity of most PTNs changed with the rhythm of tilts and of the postural corrections evoked by the tilts (Fig. 1A, B and E).

The goal of the present study was to investigate the role of the GABA_A_ inhibitory neurons of the motor cortex in the modulation of PTN posture correction-related activity. A brief account of part of this study has been published in abstract form (Tamarova *et al*., 2004).

## Materials and methods

Recordings were obtained from three adult cats, two males and a female. All experiments were conducted in accordance with NIH guidelines and were approved by the Barrow Neurological Institute Animal Care and Use Committee. Methods for behavioural training and general surgical procedures have been previously described (Beloozerova & Sirota, 1993; Beloozerova *et al*., 2005; Prilutsky *et al*., 2005). Surgery was performed under aseptic conditions using Isoflurane anesthesia (2–5%) administered by inhalation for the length of the surgery. Following the surgery, but prior to extubation, 0.005 to 0.01 mg/kg of analgesic Buprenorphine was administered intramuscularly and another dose was administered 12 h thereafter. After the extubation the cat was placed in a warm padded cage and respiration and reflexes were monitored until the cat regained conciousness.

The cat stood on a platform while continuously licking food ejected from a feeder in front of it (Fig. 1A). The platform periodically tilted in the frontal (roll) plane of the animal (Fig. 1B). A sine-like tilt trajectory with a period of 1 s and amplitude of ±15° was used. After some training, the cats effectively compensated for the platform tilts by extending limbs on the side moving down and flexing limbs on the opposite side (Beloozerova *et al*., 2005). Postural corrections helped the animal to maintain the dorsal-side-up orientation of the head and trunk and to keep the mouth against the feeder.

The neuronal activity in the forelimb representation of area 4γ of the motor cortex was recorded extracellularly using either platinum–tungsten quartz-insulated microelectrodes (40 µm outer diameter) pulled to a fine tip and mechanically sharpened (Reitboeck, 1983), or tungsten varnish-insulated electrodes (Frederick Haer & Co.). In order to identify the motor forelimb representations in each subject, three approaches have been used: (i) somatic receptive field mapping; (ii) observation of neuronal activity during voluntary movements; and (iii) intracortical microstimulation: trains of 10 50 mA cathodal pulses at 200 Hz, with each pulse of 0.2 ms duration (see Beloozerova *et al*., 2005 for details of identification procedures). A freshly pulled two- or three-barrelled pipette (glass BF-100-50-10; Sutter Instruments Co.) was glued to the recording electrode with a horizontal pipette–electrode tip separation of 15–70 µm and a vertical separation of 30–100 µm **(**Fig. 1C). Before gluing, the tip of the pipette was broken back to an inner diameter (ID) of each barrel of 2–5 µm. The horizontal distance between openings of neighbouring pipettes was 4–10 µm. The electrode–pipette assembly was inserted into the motor cortex through a hole in a plastic plate implanted above it, and advanced into the cortical tissue by a miniature manual microdrive (Fig. 1D). The criterion for identification of PTNs was the test for collision of spontaneous and antidromic spikes evoked by electrical stimulation of the pyramidal tract at the medullar level (Bishop *et al*., 1962; Fuller & Schlag, 1976).

The GABA_A_ receptor antagonists gabazine and bicuculline methiodide (both obtained from Sigma) were separately and freshly dissolved in sterile 0.9% sodium chloride with pH 3–4 at a concentration of 20 mm. We were not aiming to compare the effects of gabazine and bicuculline; rather, we used the two substances as tools to achieve GABA_A_ receptors blockade. Gabazine or bicuculline was delivered in the vicinity of the target neuron by positive currents of 50–100 nA (pipettes with ID 5 µm) or 100–200 nA (pipettes with ID 2–4 µm) applied for 50–100 s. Between the applications, a retaining negative current of 20 nA was used. In all experiments, positive current equal to the drug application current was applied for 100 s through a pipette filled with physiological solution, and changes in the activity of the neuron were monitored for 1–10 min after the application. If at any point after gabazine or bicuculline application any paroxysmal activity occurred, data were discarded, the electrode was withdrawn and the cat was returned to the home cage.

Postural tests included 50–100 tilt cycles before a GABA_A_ receptor antagonist application (control) and 300–600 tilt cycles during and immediately after the application. If needed, an additional test for recovery consisting of 100–200 tilt cycles was performed 30–40 min after the GABA_A_ antagonist application. All tilts were performed in a series of 15 cycles, which were separated by 15-s periods of stable platform. In selected experiments, applications were repeated after the activity of the neuron had returned to the control level, but no sooner than 1 h after the initial application.

**Fig. 2:**
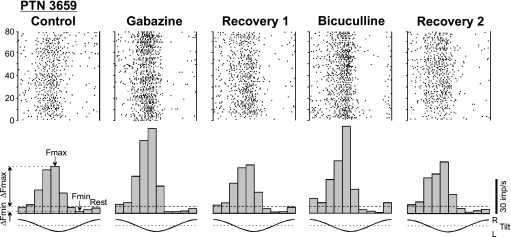
A representative example of the effect of gabazine and bicuculline on postural responses of a PTN. A raster and histogram of the PTN activity during 80 tilt cycles are shown for each of the following conditions: Control, before application; Gabazine, 2 min after gabazine application (20 mm, 100 nA, 100 s); Recovery 1 is 15 min after gabazine application; Bicuculline, 3 min after bicuculline application (20 mm, 100 nA, 100 s). Recovery 2 is 15 min after bicuculline application. Note the significant increase in peak activity in both Gabazine and Bicuculline tests while the rest, trough activity and timing of activity did not change. Fmax and Fmin, the maximum and minimum frequencies in the histogram; ΔFmax = Fmax − Frest; ΔFmin = Frest − Fmin.

For neuronal data analysis, each of the tilt cycles was divided into 10 equal bins, the peak of the right tilt being taken as the cycle onset. For each neuron, a histogram of spike activity in the tilt cycle was generated, and then the activity was averaged over all successive cycles of a given test. The following parameters were determined for each neuron: (i) the mean frequency of discharge on a stable platform (Frest); (ii) the mean frequency of discharge during tilts (Ftilt); (iii) the maximum and minimum frequency in the histogram, Fmax and Fmin; (iv) the differences between Frest and Fmax or Fmin: ΔFmax = Fmax − Frest and ΔFmin = Frest − Fmin (as shown in Fig. 2); (iv) the coefficient of modulation M = (1 − Fmin/Fmax) × 100% (the Rayleigh test for directionality was used to determine whether the activity of a neuron was modulated; Batshelet, 1981; Fisher, 1993); and (vi) the phase position of Fmax. All quantitative data are presented as the mean ± SD. The *t*-test was used to characterize the statistical significance when comparing different means; the significance level was set at *P* = 0.05.

Toward the end of experiments, several reference lesions were made, in the region of the motor cortex where neurons were sampled, by application of 30 µA DC cathodal current for 10 s through a recording electrode. At the termination of the experiment, cats were deeply anaesthetized with pentobarbital sodium. Cats were then perfused with isotonic saline followed by a 10% formalin solution. Frozen brain sections of 50 µm thickness were cut in the regions of recording and stimulating electrodes. The tissue was stained for Nissl substance with Cresyl Violet. The position of the stimulation electrode in the medullar pyramids was verified by observation of electrode track gliosis. Positions of recording tracks in the motor cortex were estimated in relation to the reference lesions.

## Results

The posture-related activity of 28 PTNs from 11 tracks through the forelimb representation of the motor cortex was tested before and after iontophoretic application of gabazine (*n* = 17) or bicuculline (*n* = 19) in the immediate vicinity of the neurons. In no instance was there any systemic behavioural effect of a GABA_A_ receptor antagonist application. The tested neurons were representative of the larger population of forelimb-related PTNs, whose posture-related activity on the tilting platform was described in a previous paper (Beloozerova *et al*., 2005). The Frest of these cells was 10.1 ± 5.3 imp/s, and the Ftilt was 12.4 ± 7.8 imp/s (not significantly different). In the control, the activity of all neurons except for one was modulated in relation to the tilt cycle, and the coefficient of frequency modulation (M) was 73 ± 18%.

Figure 2 provides a typical example of the effect of iontophoretic application of gabazine and bicuculline on the activity of one PTN. Before application (Control), neuronal activity was modulated by tilts, with the maximum in the first half of the cycle. Two minutes after gabazine application (Gabazine), the peak activity (Fmax) increased significantly while Frest, Fmin and the Fmax phase position were unchanged. Fmax returned to the control level 15 min later (Recovery 1). Three minutes after bicuculline application (Bicuculline), the Fmax of the neuron increased again to a value similar to that with gabazine; Frest, Fmin and the Fmax phase were again unchanged. Fmax returned to the control level 15 min later (Recovery 2).

**Fig. 3:**
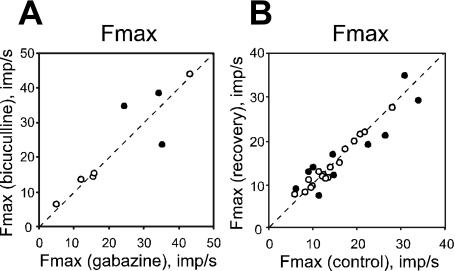
Maximum activity (Fmax) of individual PTNs under (A) Gabazine vs. Bicuculline, and (B) in Control vs. Recovery. In A and B, • denotes a PTN whose activity was statistically significantly different in the two conditions; ○, those whose activity was not significantly different.

All neurons tested with both gabazine and bicuculline (*n* = 8) responded very similarly to the two substances. Figure 3A shows Fmax in the presence of gabazine or bicuculline for each of the tested cells. The activities were very close in six out of eight cells. Similar results were obtained with each of the other parameters calculated. Therefore, for further analysis we have pooled data obtained with gabazine and bicuculline, taking the gabazine data for the cells tested with both substances. We have analysed the responses to gabazine and/or bicuculline of only those cells whose complete (*n* = 18) or clear partial (*n* = 10) recovery was documented (Fig. 3B).

**Fig. 4:**
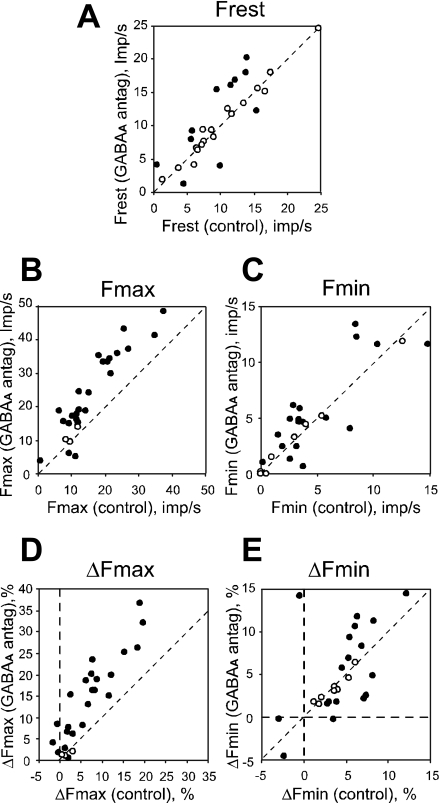
Effects of GABA_A_ receptor antagonists on the activity of individual PTNs during postural corrections. (A) Effect on the rest activity of the neurons (Frest). (B) Effect on the maximum activity of the neurons (Fmax). (C) Effect on the minimum activity of the neurons (Fmin). (D) Effect on the difference between Frest and Fmax (ΔFmax). (E) Effect on the difference between Frest and Fmin (ΔFmin). In A–E, • denotes a PTN whose activity was statistically significantly different in the two conditions; ○, those whose activity was not significantly different.

**Fig. 5:**
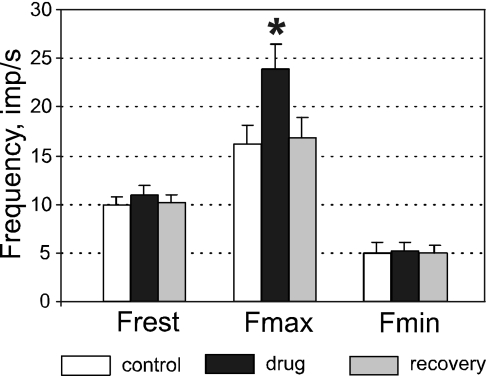
Effects of GABA_A_ receptor antagonists on PTN population activity: Frest, Fmax and Fmin. *P < 0.01 for the increase in Fmax after treatment with a GABA_A_ receptor antagonist.

After treatment with a GABA_A_ receptor antagonist, Frest did not change in 61% of cells; the activity of the remaining 39% increased (by 52 ± 22%, in 29% of cells) or decreased (by 25 ± 20%, in 10% of cells; Fig. 4A). Bicuculline caused an increase of Frest in 21% of the cells tested with it and a decrease in another 21%. The PTN population Frest stayed unchanged after treatment with a GABA_A_ receptor antagonist (Fig. 5).

In contrast to resting activity, which in many individual neurons and in the total PTN population did not change after blockade of GABA_A_ inhibition, activity during postural corrections was affected by GABA_A_ receptor antagonism in 93% (all but two) of the PTNs. The maximum effect developed in 3–8 min and lasted for ∼5 min. A significant recovery could be achieved as soon as 15 min after application and typically was complete 30–40 min thereafter. Passing a current through a pipette filled with physiological solution had no effect on any of the cells in the time window tested (1–10 min after application). A repeated application of either gabazine or bicuculline ≥1 h after the previous one gave virtually identical results.

**Fig. 6:**
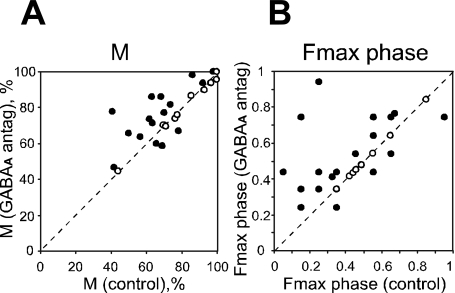
Effects of GABA_A_ receptor antagonists on (A) the coefficient of modulation and (B) the phase of maximum activity of individual PTNs. In A and B, • denotes a PTN whose activity was statistically significantly different in the two conditions; ○, those whose activity was not significantly different.

Out of 26 cells that responded to a GABA_A_ receptor antagonist during postural corrections, 21 (81%) increased their mean activity (Ftilt) by 50 ± 26%, while three cells decreased and two did not change. An even larger proportion of cells (23/26, 88%) increased their Fmax (by 9 ± 4.5 imp/s or 75 ± 50%; Fig. 4B). The same time, Fmin rose in a smaller number of cells (12/26, 46%; *P* < 0.01, *χ*^2^ test) and by a significantly smaller value (2.1 ± 1.3 imp/s; *P* < 0.05, *t*-test; Fig. 4C). Moreover, in 6 (23%) cells Fmin decreased by 4.1 ± 5.4 imp/s. The peak activity in relation to the resting level (ΔFmax) was significantly greater with a GABA_A_ receptor antagonist than in control in 22 (85%) cells (by 170 ± 130%; Fig. 4D, upper left part). In contrast, although the difference between the rest and the minimum activity (ΔFmin) was significantly altered by a GABA_A_ receptor antagonist in 18 (69%) neurons, it was smaller than in control only in half of these cells (by 73 ± 52%; lower right of Fig. 4E) and greater in the other half (by 45 ± 26%; upper left of Fig. 4E). The magnitudes of ΔFmin changes, either increases or decreases, were significantly smaller than those of ΔFmax (*P <* 0.05, *t*-test). For the whole PTN population, Fmax in the presence of a GABA_A_ receptor antagonist was significantly greater than in control, while Fmin was not different (Fig. 5). As a result of the Fmax increase (in the majority of the cells) and also the Fmin decrease (in some other cells) after GABA_A_ receptor antagonist application, the coefficient of modulation, M, rose in 13 (50%) of the PTNs (Fig. 6A).

Out of the 26 cells that responded to a GABA_A_ receptor antagonist during postural corrections, the phase of the peak activity (Fmax phase) did not change as compared to control in 8 (31%). It changed by 0.1 of a cycle in 11 (42%) cells, by 0.2 of a cycle in four (15%) cells and by >0.2 of a cycle in three (12%) cells (Fig. 6B). When Fmax changed by >0.1 of a cycle, the phase shift typically occurred because the cell became more active in a different portion of its original period of elevated activity. In only one cell did treatment with a GABA_A_ receptor antagonist lead to the appearance of a completely new activity peak; and in a different single cell, a change in the Fmax phase was the only effect of GABA_A_ receptor antagonist application.

## Discussion

We have found that the GABA_A_ receptor antagonists gabazine and bicuculline caused phase-specific changes in the posture-related activity of PTNs. Most commonly, firing frequency increased within the peak; the effects on the frequency between peaks as well as peak timing were relatively small.

Several facts suggest that in our experimental conditions the predominant action of bicuculline was the blockade of GABA_A_ receptors, rather than the blockade of Ca^2+^-dependent K^+^ current or other non-GABAergic effects. First, the resting activity of less than a quarter (21%) of PTNs increased after treatment with bicuculline. Second, across the cycle of posture-related corrections, bicuculline affected PTN responses not uniformly but in a strong phase-dependent manner, being maximal in the period of higher PTN activity. Third, in seven neurons we tested the effect of bicuculline during two different behaviours (posture maintenance and scratching or posture maintenance and locomotion). In five of these cells we observed a clear-cut effect of bicuculline on activity during one behaviour but not the other (I. Beloozerova, unpublished observation). Fourth, in eight neurons we compared the posture-related effects of bicuculline with those of another GABA_A_ receptor antagonist, gabazine, and found them to be very similar (Figs 2 and 3B). Finally, with our application regimen only rarely (in <10% of all applications) did we observe paroxysmal activity in the neurons; these data have been excluded from the analysis.

In many previous studies similar to our experiments, GABA_A_-mediated influences have been found to decrease the amplitude of neuronal responses to stimulation or elevate the threshold for the responses (e.g. Dykes *et al*., 1984; Alloway & Burton, 1991; Jacobs & Donoghue, 1991; Matsumura *et al*., 1992; Kyriazi *et al*., 1996; Sato *et al*., 1995; Sawaguchi, 2001; Li *et al*., 2002). Studies have also shown in different compartments of the sensory cortex (somatosensory, visual and auditory) and in different animal models that GABA_A_-mediated influences inhibit responses to sensory stimulation, with strong effects within many neurons' primary receptive fields (e.g. Alloway *et al*., 1989; Ferster, 1986; Borg-Graham *et al*., 1998; Moore & Nelson, 1998; Martinez *et al*., 2002; Wang *et al*., 2002; Qin & Sato, 2004). A variety of other studies have also documented a positive correlation between levels of inhibition and excitation in the cortex (e.g. Haider *et al*., 2006; Weliky *et al*., 1995; Sato *et al*., 1996; Varela *et al*., 1999; Shu *et al*., 2003; Marino *et al*., 2005; Higley & Contreras, 2006). The possible role of reducing the neuronal responses at their focus in respect of different physiological functions has been discussed in the above and other reports. It is suggested that the dynamic balance of excitatory and inhibitory interactions may confer significant computational and plasticity advantages. Regarding the role of GABA_A_-mediated inhibition in the posture-related activity of PTNs, we suggest the following. We have previously proposed that the posture-related activity of PTNs provides a control signal for postural adjustments (Beloozerova *et al*., 2005). Here we report that cortical GABA_A_-mediated influences reduce this signal. This reduction might be related to a cortical mechanism for adaptive modulation of the control signal if the need arises. During complex motor behaviours, such as walking on an unstable surface, the phase-dependent corrective control signal to the limbs can be increased by tonic inhibition of GABA_A_ergic influences on PTNs.

In our experiments, postural responses in the motor cortex after treatment with a GABA_A_ receptor antagonist increased to a lesser degree than those reported by Matsumura *et al*. (1992) for a press–release task in monkeys (75 ± 50% vs. 300 ± 150%). This is unlikely to be because we did not apply a sufficient amount of GABA_A_ receptor antagonist or because there was still significant room to increase the response by delivering more substance. We used a much stronger current and longer application time than did Matsumura *et al*. (1992). The effect of application in our experiment also lasted much longer, suggesting that a larger amount of GABA_A_ receptor antagonist was applied. It seems likely that GABA_A_-mediated influences play different roles in different behaviours and in different neuronal networks. Other authors have also reported considerable differences in the effects of a GABA_A_ receptor blockade between individual neurons and between neuronal networks (e.g. Sillito, 1975; Alloway & Burton, 1991; Matsumura *et al*., 1992; Sato *et al*., 1996; Murthy & Humphrey, 1999; Schiller & Tehovnik, 2003). For example, Alloway & Burton (1991) found fewer inhibitory effects in the slowly adapting neurons in the somatosensory cortex than in the rapidly adapting ones. Matsumura *et al*. (1992) found that the effects in the premotor cortex were weaker than those in the primary motor cortex.

During postural corrections, the interburst activity of PTNs was rather mildly and inconsistently affected by GABA_A_ receptor antagonism. Thus, the troughs in the posture-related responses of PTNs for the most part were not caused by the cortical inhibitory GABAergic interneurons that are active during these troughs. GABA_A_-mediated input to PTNs seems to play a rather small role in causing their periods of lowered activity. This finding suggests that the main cause of the periods of reduced PTN activity is a decrease of the excitatory (most probably thalamic) drive.

In addition to the main effect (an increase in the peak discharge), treatment with a GABA_A_ receptor antagonist caused a decrease in the trough activity in 23% of PTNs (Fig. 4C). Also, the peak activity decreased in two cells (Fig. 4B). These decreases in activity can be explained by the involvement of non-GABA_A_ergic inhibitory neurons that synapse on PTNs and whose activity increased (disinhibited) after treatment with a GABA_A_ receptor antagonist. With our application regimen, the GABA_A_ antagonists could affect tissue several hundreds of microns in diameter (Jacobs & Donoghue, 1991). Indeed, in few cases when we recorded two cells simultaneously with the same electrode, the GABA_A_ receptor antagonists typically affected the activity of both of them. In future studies, it would be interesting to continue examining the effects of a GABA_A_ transmission blockade on the activity of different identified classes of cortical neurons.

In this study, the phase position of the peak activity changed slightly (by 0.1 of a cycle) or not at all in the majority of PTNs (73%) after treatment with a GABA_A_ receptor antagonist. In only two neurons did the peak phase change substantially (Fig. 6B). The relatively small changes in the neuronal response timing and trough activity suggest that some source other than the cortical GABA_A_ergic mechanisms, such as thalamic or cortico-cortical inputs, largely determine the basic pattern of PTN activity during postural corrections. In contrast to sensory cortices where the responses to sensory stimuli are ‘shaped’ in different domains by intracortical GABA_A_ergic inhibition (e.g. Sillito, 1975; Dykes *et al*., 1984; Eysel, 1992; Kyriazi *et al*., 1996; Sato *et al*., 1996; Pernberg *et al*., 1998; Foeller *et al*., 2001; Tremere *et al*., 2001; Wang *et al*., 2002; Dehner *et al*., 2004; Chang *et al*., 2005), responses in the motor cortex are primarily ‘scaled’ while their timing is determined by input signals.

## Abbreviations

Fmax: maximum frequency in the histogram
Fmin: minimum frequency in the histogram
Frest: mean frequency of discharge on a stable platform
Ftilt: mean frequency of discharge during tilts
GABA: gamma-aminobutyric acid
M: coefficient of modulation
PTN: pyramidal tract neuron.
